# Association of Cigarette Smoking, COPD, and Lung Cancer With Expression of SARS-CoV-2 Entry Genes in Human Airway Epithelial Cells

**DOI:** 10.3389/fmed.2020.619453

**Published:** 2020-12-04

**Authors:** Junping Yin, Brigitte Kasper, Frank Petersen, Xinhua Yu

**Affiliations:** Division of Pulmonary Immune Diseases, Department of Asthma and Allergy, Research Center Borstel, Leibniz Lung Center, Borstel, Germany

**Keywords:** COVID-19, SARS-CoV-2, human airway epithelial cells, ACE2 (angiotensin converting enzyme-2), smoking, transmembrane serine protease 2, cathpesin L

## Abstract

SARS-CoV-2 enters into human airway epithelial cells via membrane fusion or endocytosis, and this process is dependent on ACE2, TMPRSS2, and cathepsin L. In this study, we examined the expression profiles of the three SARS-CoV-2 entry genes in primary human airway epithelial cells isolated from smokers, non-smokers, patients with chronic obstructive pulmonary disease or lung cancer. An exhaustive search of the GEO database was performed to identify eligible data on 1st June 2020. In total, 46 GEO datasets comprising transcriptomic data of 3,053 samples were identified as eligible data for further analysis. All meta-analysis were performed using RStudio. Standardized mean difference was utilized to assess the effect size of a factor on the expression of targeted genes and 95% confidence intervals (CIs) were calculated. This study revealed that (i) cigarette smoking is associated with an increased expression of ACE2 and TMPRSS2 and a decreased expression of cathepsin L; (ii) significant alternations in expression of ACE2, TMPRSS2, and cathepsin L were observed between current smokers and former smokers, but not between former smokers and never smokers; (iii) when compared with healthy controls with identical smoking status, patients with COPD or lung cancer showed negligible changes in expression of ACE2, TMPRSS2, and cathepsin L. Therefore, this study implicates cigarette smoking might contribute to the development of COVID-19 by affecting the expression of SARS-CoV-2 entry genes, while smoking cessation could be effective to reduce the potential risk.

## Introduction

In December 2019, a cluster of viral pneumonia cases which is featured by pulmonary parenchymal opacities at chest radiography was reported in Wuhan, China (1). Analysis of lower respiratory tract samples from patients identified a novel beta-coronavirus termed severe acute respiratory syndrome coronavirus 2 (SARS-CoV-2) as the casual pathogen for the pneumonia (2). On 11 March 2020, the World Health Organization (WHO) declared coronavirus disease 2019 (COVID-19) as a pandemic (3). As of Oct 15, 2020, there have been over 40 million laboratory-confirmed cases of COVID19 worldwide with more than one million deaths (4). SARS-CoV-2 shares ~80% sequence identify to SARS-CoV and both viruses use the same cell entry receptor, namely angiotensin converting enzyme 2 (ACE2) (5, 6). As the receptor of SARS-CoV and SARS-CoV-2, ACE2 mediates the viral entry via two major pathways, cathpesin L-dependent endocytosis and transmembrane serine protease 2 (TMPRSS2) dependent membrane fusion (7–9). Since COVID-19 is an acute disease resulted from respiratory tract infection of SARS-CoV-2, the interaction between the spike protein (S protein) of the virus and the ACE2 on human airway epithelial cells could be a crucial step for the development of the disease (10, 11).

Although SARS-CoV-2 can infect individuals of any age, most of the severe cases have been reported in older adults or patients with significant comorbidities including chronic respiratory diseases (12). For example, a study with large case series reported a higher prevalence of COPD in patients with severe COVID-19 (12), and this finding was confirmed by several subsequent studies (13–15). Meta-analysis revealed that COPD is associated with a significant, roughly 5-fold increased risk for the development of severe COVID-19 infections (16, 17). Understanding the molecular mechanisms behind the increased risk for severe COVID-19 in patients with chronic respiratory diseases would provide clues toward their pathophysiology and the identification of therapeutic targets. Since ACE2 might play an important role in protecting the host against lung injury (18), one hypothesis is that the expression of ACE2 in airway epithelial cells of patients with chronic respiratory diseases might be upregulated, which enhances the infection of SARS-CoV-2. This notion is partially supported by a recent study, in which Leung et al. reported that smokers and patients with COPD show a higher levels ACE 2 than healthy non-smokers (19).

In this study, our objective is to explore the effect of on the expression of three genes essentially involved in SARS-CoV-2 entry, namely ACE2, TMPRSS2, and cathepsin L in human airway epithelial cells isolated from non-smoker, smoker, and patients with cigarette smoking (CS)-induced diseases. To reach this aim, we retrieved previously published transcriptomic datasets of human airway epithelial cells of healthy subjects with different status of cigarette smoking as well as patients with COPD or lung cancer.

## Materials and Methods

### Data Retrieval

**Figure 1:**
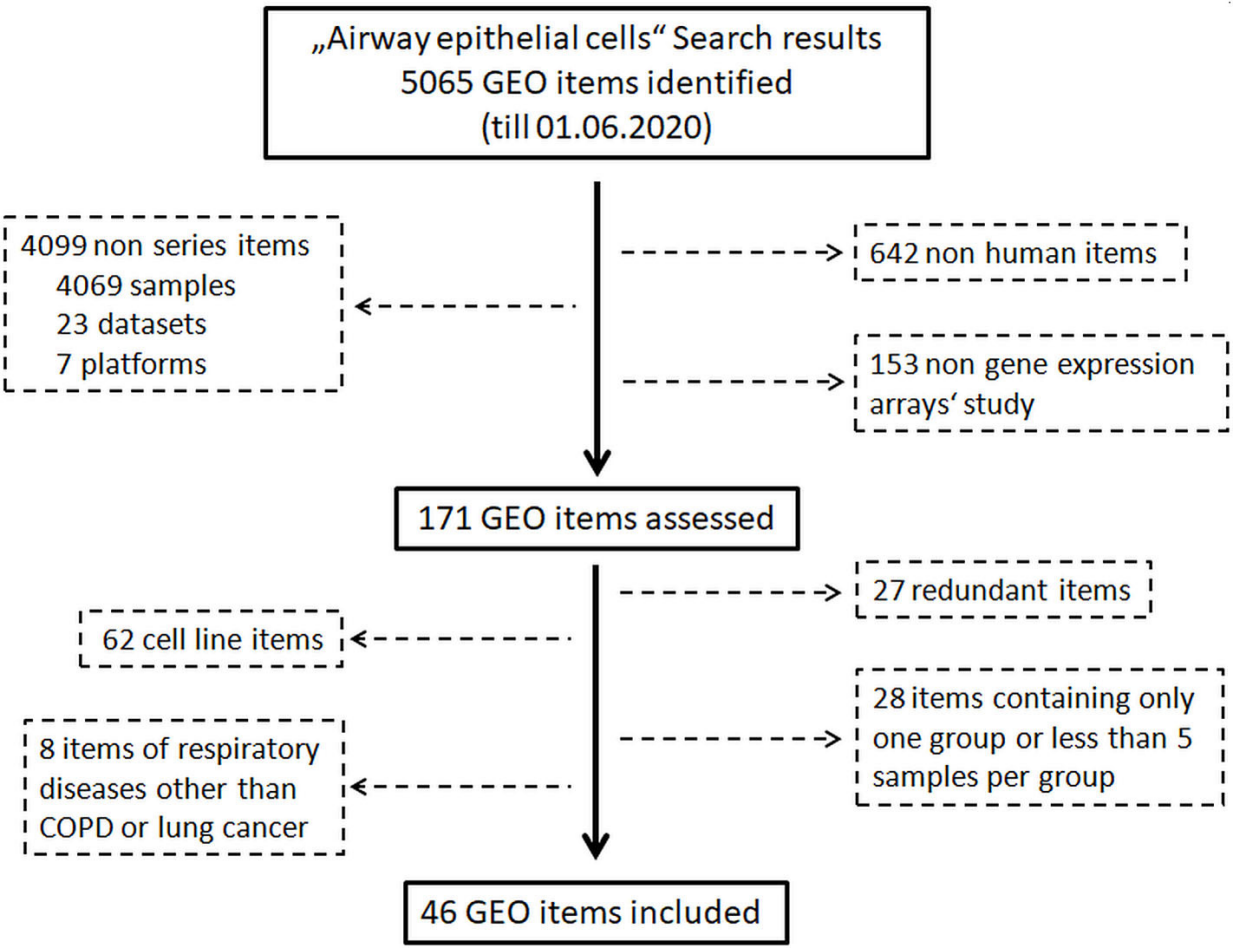
Flow diagram of GEO databases screening and selection process.

An exhaustive search of the Gene Expression Omnibus (GEO) database (https://www.ncbi.nlm.nih.gov/geo/) was performed to identify eligible data on 1st June 2020. The procedure of identification of eligible data is shown in Figure 1. First of all, the key word “Airway epithelial cells” was used for the search without any limitations. In a second step, non-human datasets, non-series datasets, and non-gene expression array datasets were filtered out. Finally, datasets from step two were reviewed carefully, and the following datasets were excluded: (a) datasets of cell line(s), (b) redundant dataset, (c) datasets containing only one group, (d) datasets of respiratory diseases other than COPD and lung cancer, and (e) datasets containing <5 samples per group. Healthy groups consisted of asymptomatic subjects with normal pulmonary functions, while pathological conditions including COPD and lung cancer were defined using criteria described in original studies.

### Data Correction, Normalization

For affymetrix microarrays, CEL files were uploaded into RStudio (Version 1.3.959, based on R version 4.0.1) using package “affy” (20). Subsequently, background correction and normalization were applied to the raw data using “Robust Multichip Average (RMA)” method (21). For agilent microarray data, raw expression profiling files were uploaded into RStudio using package “limma” (22). By using the “limma” package, background correction and normalization were performed using “normexp” and “quantile” methods, respectively. Background corrected, normalized and log2 transformed signal intensities of ACE2, TMPRSS2, and cathepsin L were outputted and used for further analysis.

### Meta-Analysis

All meta-analysis were performed using “meta” package in RStudio. Standardized mean difference (SMD) was utilized to assess the effect size of a factor on the expression of targeted genes, and 95% confidence intervals (CIs) of SMD were calculated (23). According to the guideline proposed by Cohen (24), the magnitude of the SMD is interpreted as below: small, SMD = 0.2; medium, SMD = 0.5; and large, SMD = 0.8. To evaluate the influence of pack-years of smoking in current smokers on the gene expression, meta-analysis was performed to determined correlation coefficients and *p*-values. Fixed or random effect model was applied to pool the effect size depending on the heterogeneity across the datasets determined by inconsistency (*I*^2^) statistics and Cochrane's Q test. random effect model was applied when there was significant heterogeneity among datasets (*I*^2^ value > 50% or *P*-value of Q-test < 0.05), otherwise fixed effect model was utilized (25, 26).

### Statistics

All statistical analyses were conducted using RStudio. Statistical significance between two groups was calculated using Student's *t*-test or Mann Whitney U-test depending on the normality test of the data. Linear regression model was generated to evaluate the correlation of age or lung function index of COPD patients and healthy controls with gene expression of SARS-CoV-2 entry genes. To assess the correlation between pulmonary function parameters and gene expression, multiple linear regression was used and age was added as a covariate. A *P* < 0.05 was considered as statistical significance.

## Results

### Study Selection and Data Retrieval

A search of the Gene Expression Omnibus (GEO) database with the key word “Airway epithelial cells” resulted in 5,065 hits. In the next step, 642 non-human items, 4,099 non-series items, and 153 items of non-gene expression array were filtered out. Subsequent assessment further excluded 125 items which were transcriptomic data of cell lines, redundant data, other respiratory diseases or datasets containing only one group or <5 samples per group. Finally, 46 GEO datasets comprising 3,053 transcriptomic data of human airway epithelial cells were identified (Figure 1, Supplementary Figure 1). Microarray data of each dataset were retrieved from the GEO database. After combining datasets generated by same research group with identical platforms, 20 transcriptomic datasets were generated and used for further analysis (Supplementary Table 1). After background correction and normalization of each transcriptomic dataset, expression levels of ACE2, TMPRSS2, and cathepsin L were used for further analysis.

### Effect of Age, Gender, and Cell Type on Expression of ACE2, TMPRSS2, and Cathepsin L

Before proceeding to the comprehensive meta-analysis, we evaluated the effect of age, gender and cell type on the expression of the three SARS-CoV-2 entry genes. As shown in Supplementary Figure 2, no significant correlation was observed between age and gene expression of ACE2, TMPRSS2, and cathepsin L in healthy smokers or patients, with an exception of a weak correlation between age and expression of cathepsin L in patients with lung cancer. Negligible association was also observed between gender and expression of the three SARS-CoV-2 entry genes (Supplementary Figure 3). In contrast to age and gender, cell type showed a great impact on expression of ACE2 and TMPRSS2. As shown Supplementary Figure 4, trachea airway epithelial cells (TAEC) expressed significantly higher levels of ACE2 and TMPRSS2 than both small airway epithelial cells (SAEC) and large airway epithelial cells (LAEC) (Supplementary Figure 4).

### Expression of ACE2, **TMPRSS2, and Cathepsin L in Never Smoker, Former Smoker, and Current Smokers**

**Figure 2:**
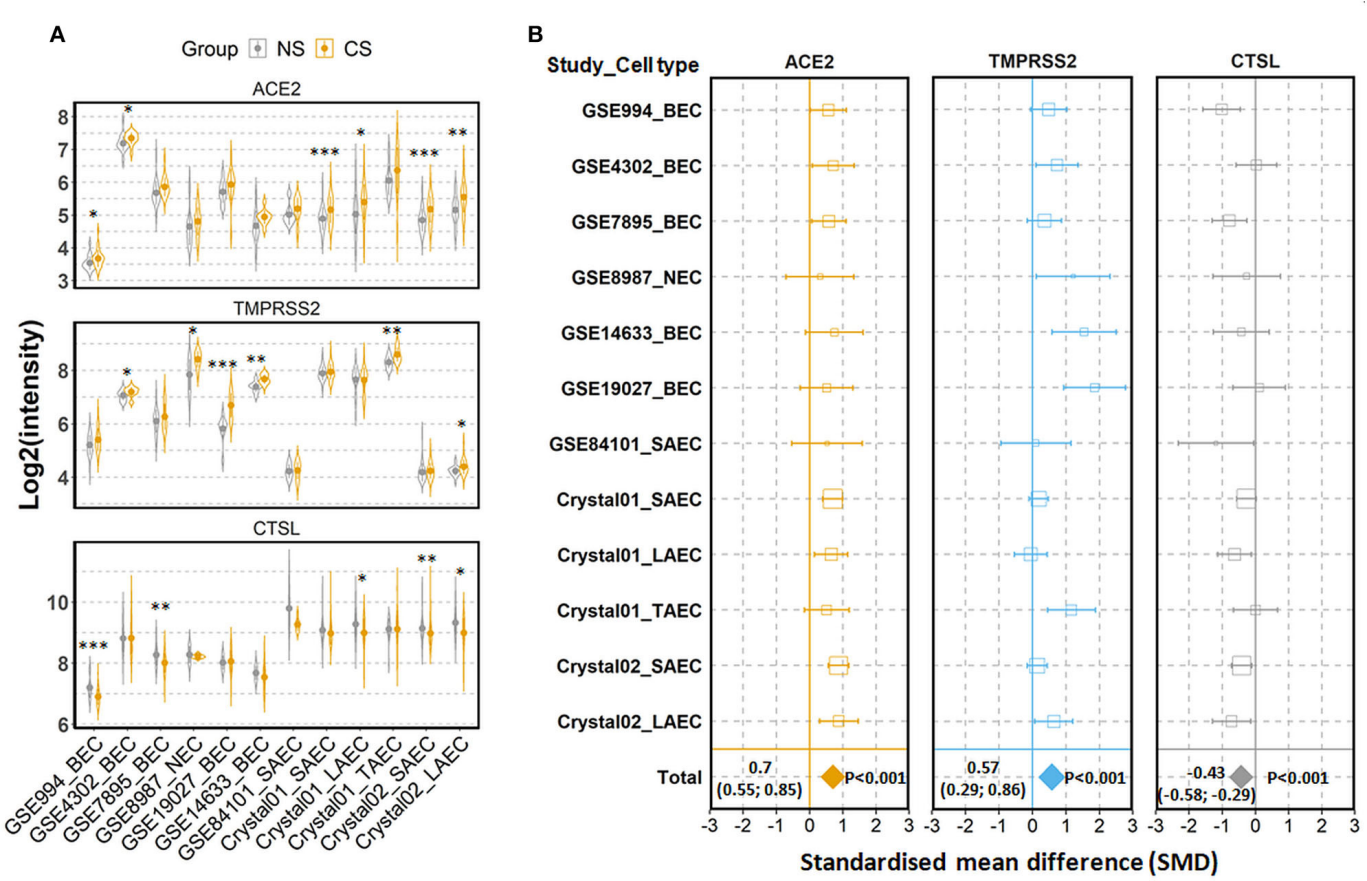
Expression of ACE2, TMPRSS2, and cathepsin L (CTSL) in airway epithelial cells of healthy current smokers (CS) and never smokers (NS). **(A)** Violin plot of expression levels of ACE2, TMPRSS2, and CTSL in 12 datasets which contain current smokers and never smoker. Mean and standard deviation (SD) of each group are presented as dot and line, respectively. Statistical difference was calculated by Student's *t*-test. *p < 0.05, **p < 0.01, ***p < 0.001. **(B)** Forest plot of 12 datasets examining expression of ACE2, TMPRSS2, and CTSL in current smokers and never smokers. The x-axis indicates the standardized mean difference (SMD), while the y-axis shows GEO datasets and cell types. Each square in the plots represents the SMD in corresponding datasets and the 95% confidence interval (CI) is shown by the error bar. The size of each square represents the weight of the individual dataset in the meta-analysis. The diamonds in the bottom represent the SMD of the meta-analysis. The SMD, 95% CI and *P*-values of meta-analysis are depicted. BEC, bronchial epithelial cell; NEC, nasal epithelial cell; SAEC, small airway epithelial cell; LAEC, large airway epithelial cell; TAEC, trachea airway epithelial cell.

To examine the expression of the three genes in airway epithelial cells under physiological conditions, we performed comparisons among healthy individuals according to their status of cigarette smoking. The first comparison was performed between current smokers and never smokers. In total, 12 comparisons were performed, with 594 current smokers and 329 never smokers (27–43). As shown in Figure 2A, mean values of expression levels of AEC2 in current smoker were consistently higher than those in never smokers in all 12 comparisons, and differences in 6 out of 12 were statistically significant. Meta-analysis for the standardized mean difference (SMD) showed a moderate effect of smoking on ACE2 expression (SMD = 0.70, *P* < 0.001) (Figure 2B). Similar to ACE2 expression, the expression of TMPRSS2 was also significantly increased in airway epithelial cells of current smoker as compared to never smokers (SMD = 0.57, *P* = 0.001) (Figures 2A,B). By contrast, smoking affected the expression of cathepsin L in the opposite direction. As compared to never smokers, current smokers expressed significantly lower levels of cathepsin L (SMD = −0.43, *P* < 0.001) (Figures 2A,B).

**Figure 3:**
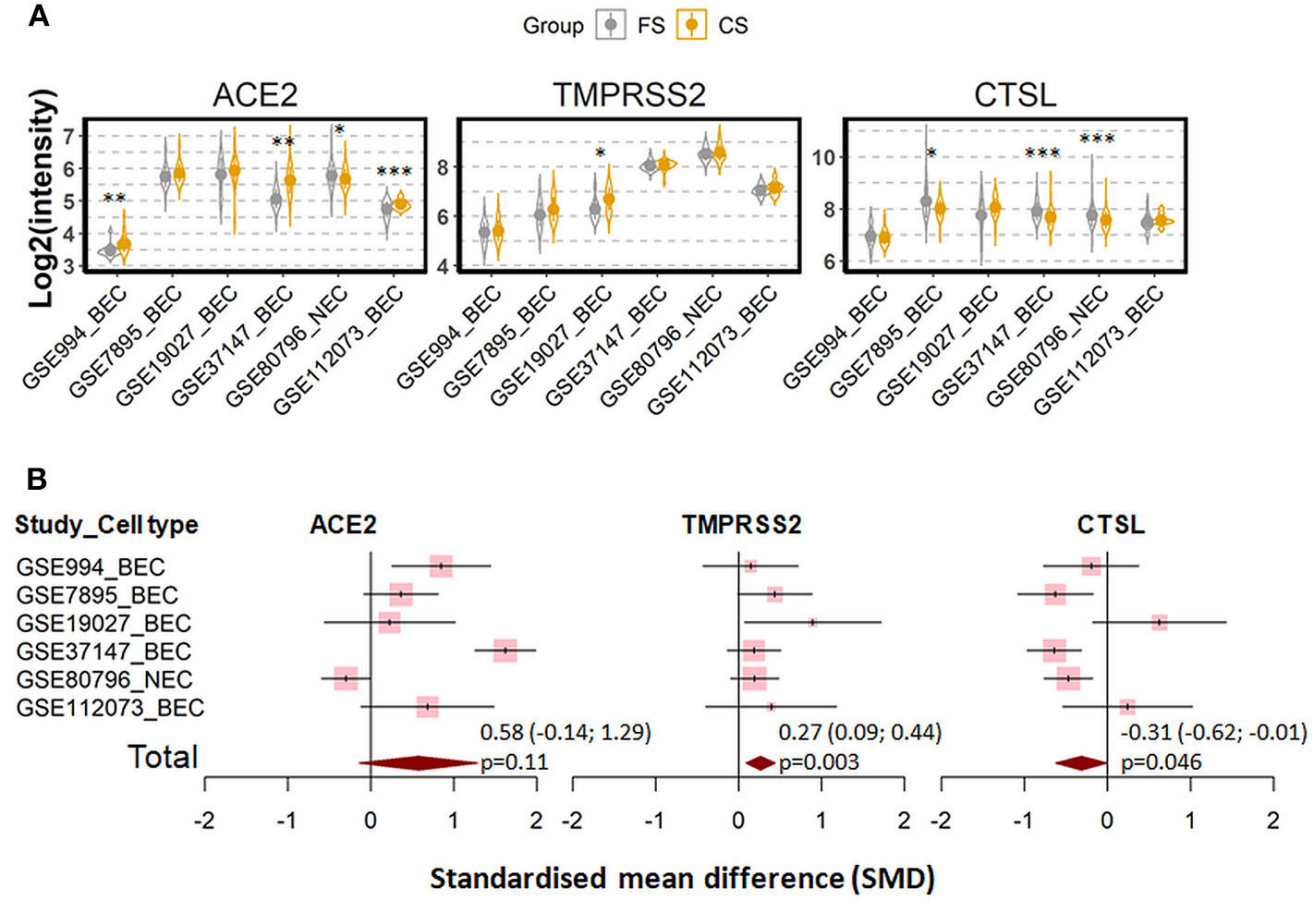
Expression of ACE2, TMRPSS2, and cathepsin L (CTSL) in airway epithelial cells of healthy current smokers (CS) and former smokers (FS). **(A)** Violin plot of expression levels of ACE2, TMPRSS2, and CTSL in six datasets which contain current smokers and former smoker. The mean and standard deviation (SD) of each group were presented as dot and line, respectively. BEC, bronchial epithelial cells; NEC, nasal epithelial cells. Statistical differences were calculated by Student's *t*-test. *p < 0.05, **p < 0.01, ***p < 0.001. **(B)** Forest plot of six datasets examining expression of ACE2, TMPRSS2, and CTSL in current smokers and former smokers. The x-axis indicates the standardized mean difference (SMD), while the y-axis shows GEO datasets and cell types. Each square in the plots represents the SMD in corresponding datasets and the 95% confidence interval (CI) is showed by the error bar. The size of each square represents the weight of the individual dataset in the meta-analysis. The diamonds in the bottom represent the SMD of the meta-analysis. The SMD, 95% CI and *P*-values of meta-analysis are depicted.

**Figure 4:**
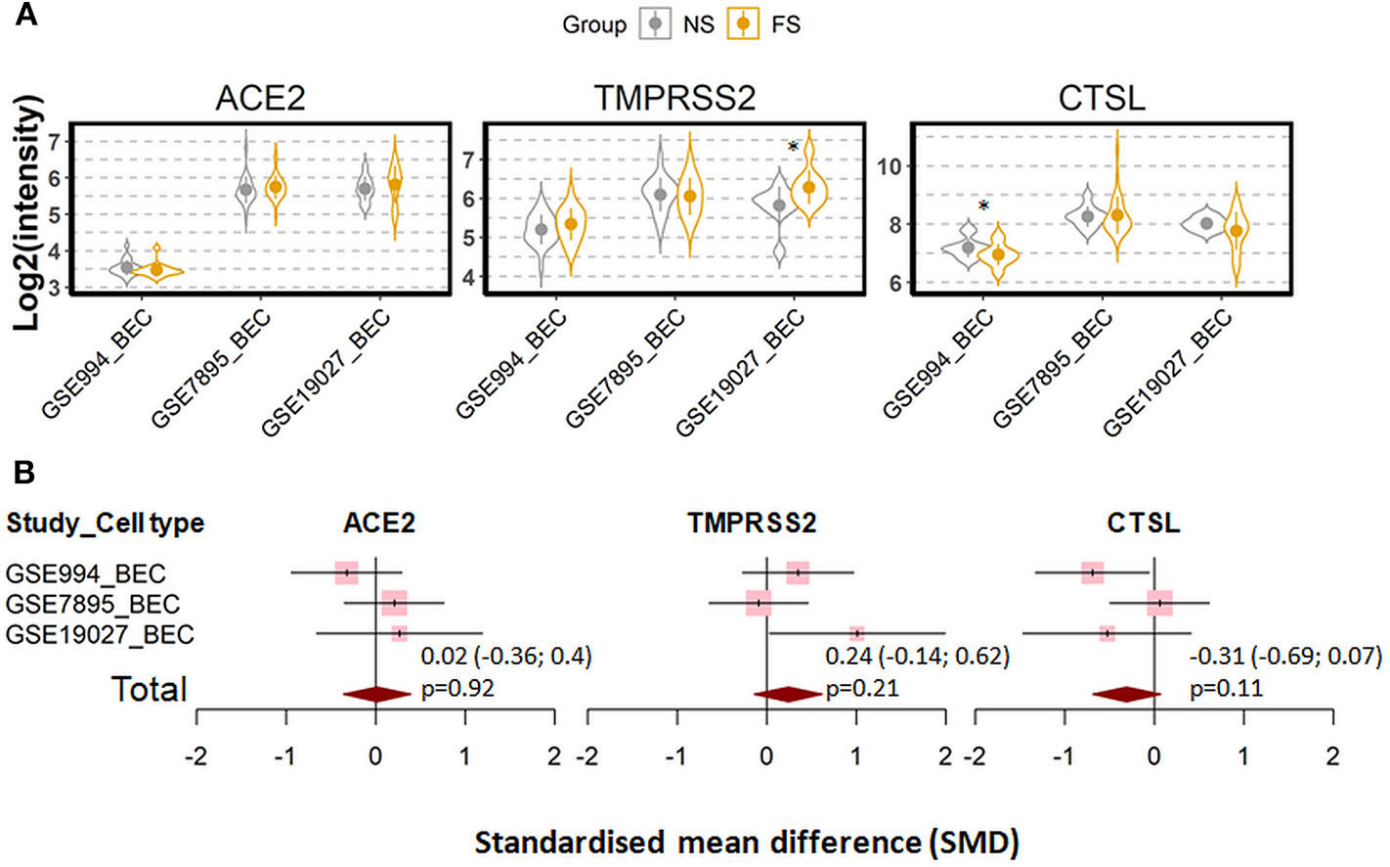
Expression of ACE2, TMRPSS2 and cathepsin L (CTSL) in the airway epithelial cells of healthy never smokers (NS) and former smokers (FS). **(A)** Violin plot of expression levels of ACE2, TMPRSS2, and CTSL in three datasets which contain current smokers and former smoker. BEC, bronchial epithelial cells. Statistical difference was calculated by Student's *t*-test. *p < 0.05. **(B)** Forest plot of three datasets examining expression of ACE2, TMPRSS2, and CTSL in never smokers and former smokers. The x-axis indicates the standardized mean difference (SMD), while the y-axis shows GEO datasets and cell types. The SMD, 95% CI and *P*-values of meta-analysis are depicted.

We next investigated whether the effect of cigarette smoking on the three genes reflects a chronic or an acute response of the tissue. Six datasets which contain expression data of former smokers and current smokers were recruited for this comparison (28, 35, 41, 44–46). Notably, the differences in the expression of the three genes between current smokers and former smokers follow the same pattern observed between current smokers and never smokers. As compared to former smokers, current smokers showed a moderate increased expression levels of ACE2 and TMPRSS2, which was found to be significant only for the latter protein (ACE2; SMD = 0.58, *P* = 0.11 and TMPRSS2; SMD = 0.27, *P* = 0.003, respectively). By contrast, decreased levels were seen for cathepsin L (SMD = −0.31, *P* = 0.046) (Figure 3). In line with this observation, a comparison between never smokers and former smokers (28, 35, 41) revealed no significant differences between these group with regard to the expression of these genes (Figure 4).

Thus, these results suggest that the effect of cigarette smoking on expression of ACE2, TMPRSS2, and cathepsin L is more likely an acute reaction than a chronic alteration of the tissue. The acute effect of cigarette smoking on ACE2 was confirmed in a further dataset (47). In this study, current smokers were asked to refrain from cigarette smoking for at least 2 days and then subjected to acute smoking exposure. As shown in Supplementary Figure 5, the acute smoking exposure increased the expression of ACE2, but showed no effect on TMPRSS2 or cathepsin L.

To further examine the effect of cigarette smoking on expression of SARS-CoV-2 entry genes, we next determined whether smoking intensity was correlated with expression of ACE2, TMPRSS2 and cathepsin L. Meta-analysis with seven datasets showed that there was no significant correlation between the pack-years of smoking and expression of any of the three genes (Supplementary Figure 6).

### Expression of ACE2, TMPRSS2, and Cathepsin L in Patients With COPD

Consequently, we compared in the next step expression profiles of the three SARS-CoV-2 entry genes of epithelial cells derived from none-diseased subjects with those of patients with different respiratory disorders. Since smoking has a major impact on the expression of these genes, we focused on COPD as a smoking-related disease in a first approach.

**Figure 5:**
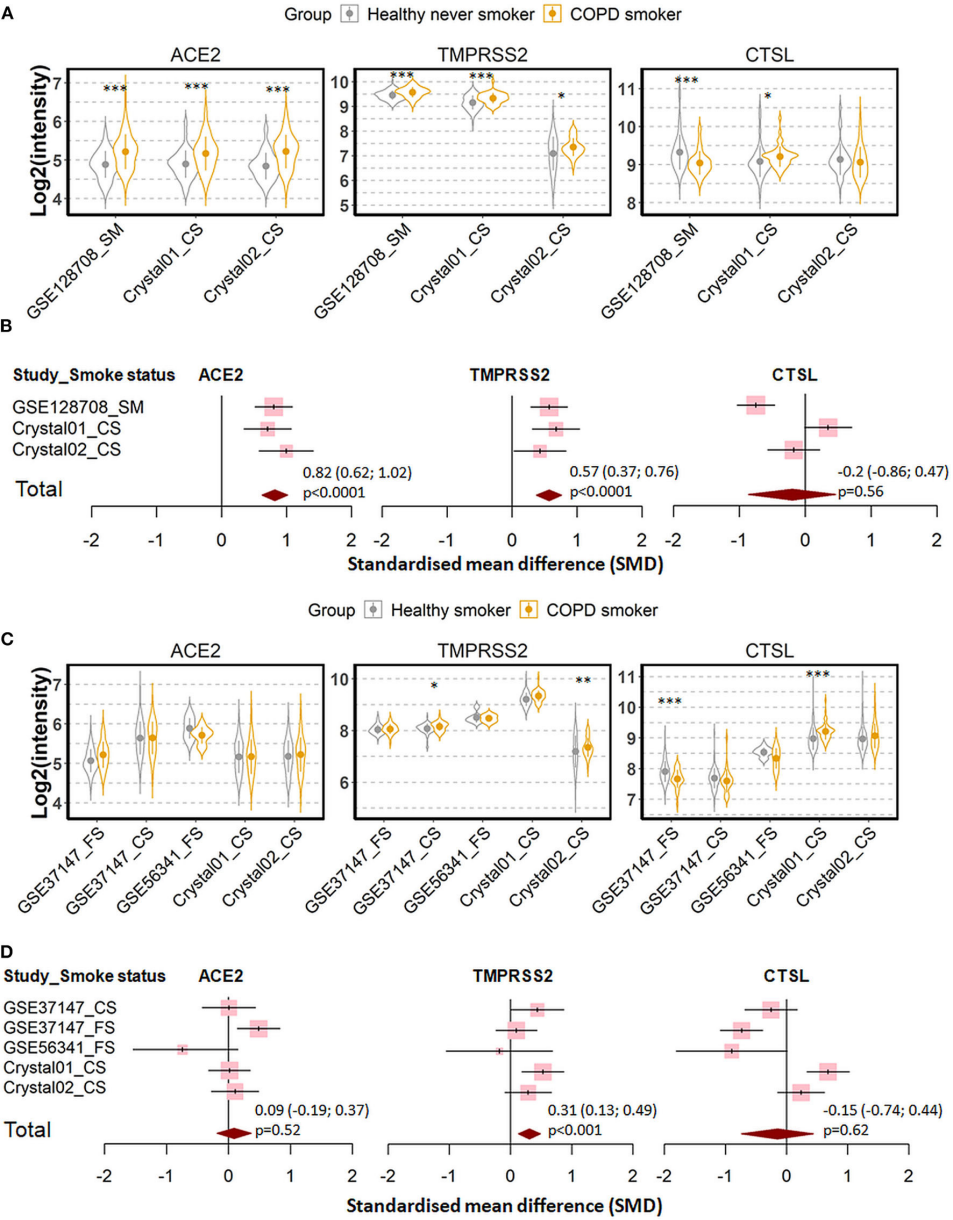
Expression of ACE2, TMPRSS2, and cathepsin L (CTSL) in airway epithelial cells of healthy subjects and COPD patients. **(A)** Violin plot of expression levels of ACE2, TMPRSS2, and CTSL in three datasets which contain healthy never smokers and patients with COPD. Statistical difference was calculated by Student's *t*-test. *p < 0.05, **p < 0.01, ***p < 0.001. **(B)** Forest plot of three datasets examining expression of ACE2, TMPRSS2, and CTSL in healthy never smokers and patients with COPD. The SMD, 95% CI and *P* values of meta-analysis are depicted. **(C)** Violin plot of expression levels of ACE2, TMPRSS2, and CTSL in five datasets which contain healthy smokers and patients with COPD. **(D)** Forest plot of five datasets examining expression of ACE2, TMPRSS2, and CTSL in healthy smokers and patients with COPD. CS, Current smokers; FS, Former smokers.

Three datasets were recruited to compare never smoker control and COPD patients (27, 29–33, 37, 38, 40, 43, 48). Expression of ACE2 and TMPRSS2 were found to be increased, while the expression of cathepsin L was decreased in COPD patients as compared to never smoker controls (Figures 5A,B). Meta-analysis revealed a strong effect of COPD on ACE2 expression (SMD = 0.82, *P* < 0.0001), a moderate effect on TMPRSS2 (SMD = 0.57, *P* < 0.0001), and no significant effect on cathepsin L.

Since most the COPD patients were smokers, we next determined whether the effect of COPD on expression of our target genes is due to cigarette smoking by comparing COPD patients with healthy smokers (27, 29–33, 37, 38, 40, 43, 46, 49). As shown in Figures 5C,D, expression levels of ACE2 and cathepsin L are similar between both groups. The only difference between the two groups could be assigned to the expression of TMPRSS2, where COPD patients showed moderately higher RNA levels than healthy smokers (SMD = 0.31, *P* < 0.001). According to these results, the observed difference in the expression of the three SARS-CoV-2 entry genes between COPD patients and healthy controls is mainly due to cigarette smoking but not to disease manifestation.

To substantiate these findings, we examined the relationship between the lung function of COPD patients and gene expression by data from a further study in which lung function parameters were included (46). Expression levels of ACE2, TMPRSS2 and cathepsin L were not correlated with the first second of forced expiration (FEV1) or FEV1/FVC (Forced vital capacity) suggesting that the impairment of lung function does not affect the expression SARS-CoV-2 entry genes (Supplementary Figure 7).

### Expression of ACE2, TMPRSS2, and Cathepsin L in Patients With Lung Cancer

**Figure 6:**
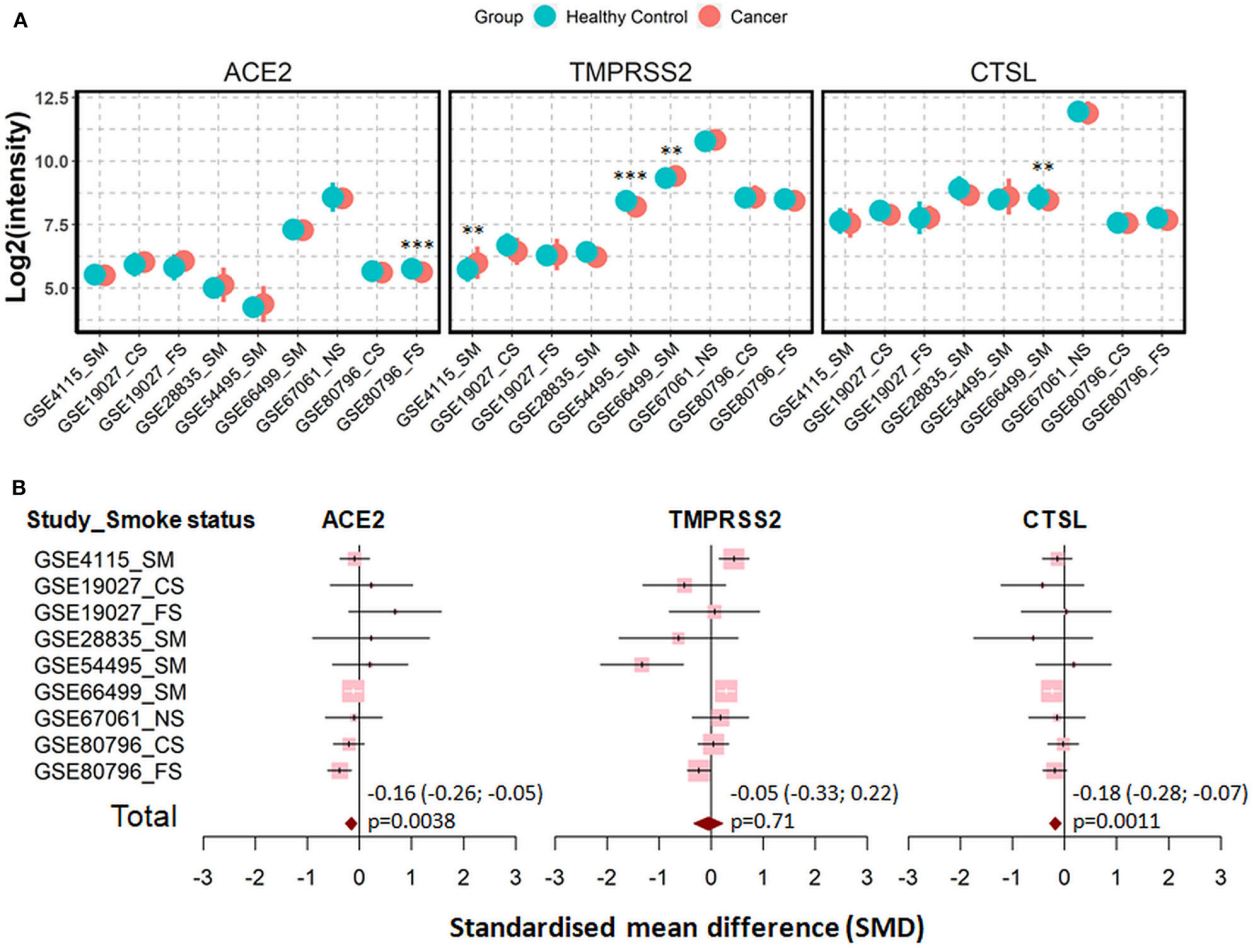
Expression of ACE2, TMRPSS2, and cathepsin L (CTSL) in airway epithelial cells of healthy controls and patients with lung cancer. **(A)** Plot of expression levels of ACE2, TMPRSS2, and CTSL in nine datasets which contain healthy control and patients with lung cancers. Statistical difference was calculated by Student's *t*-test. **p < 0.01, ***p < 0.001. **(B)** Forest plot of nine datasets examining expression of ACE2, TMPRSS2, and CTSL in healthy controls and patients with lung cancer. The x-axis indicates the standardized mean difference (SMD), while the y-axis shows GEO datasets and cell types. The SMD, 95% CI and *p*-values of meta-analysis are depicted. NS, never smoker; CS, Current smokers; FS, Former smokers; SM, Smokers.

Cigarette smoking does not only promote COPD but represents also a main cause for lung cancer. Therefore, we evaluated next the expression the three genes in patients with lung cancer and in corresponding healthy controls. In total, seven datasets were recruited for the comparison, including 1,003 patients and 540 healthy controls (41, 44, 50–54). Of note, healthy control subjects and patients in each dataset were identical in smoking status, either never smokers, former smokers or current smokers. Meta-analysis showed that lung cancer is associated with a very mild effect on modulation of expression of ACE2 (SMD = −0.16, *P* = 0.0038) and cathepsin L (SMD = −0.18, *P* = 0.0011) (Figure 6).

Finally, we determined whether types of cancers and cancer-related mutation affected the expression of SARS-CoV-2 entry genes. No significant difference in expression of ACE2, TMPRSS2, or cathepsin L between patients with adenocarcinoma and squamous cell carcinoma (Supplementary Figure 8), suggesting types of lung cancer show no effect on expression of SARS-CoV-2 entry genes. However, cancer-related mutations affected expression of SARS-CoV-2 entry genes in adenocarcinoma patients from a previous study (55), where the Kirsten Rat Sarcoma virus (*KRAS*) mutation was associated with slight but significant decrease in expression of ACE2 and TMPRSS2, and the epidermal growth factor receptor (*EGFR*) mutation was associated with increased expression of ACE2 and TMPRSS2 and decreased expression of cathepsin L (Supplementary Figure 9).

## Discussion

In this study, we examined expression levels of ACE2, TMPRSS2, and cathepsin L in human airway epithelial cells derived from never smokers, former smokers, current smokers, patients with COPD, or lung cancer. By performing comprehensive meta-analysis, we generated a complete picture of expression profiling of the three SARS-CoV-2 entry genes.

In our current study we could demonstrate that current smokers show a highly significant increase in ACE2 expression as compared to never smokers, which is in line with previous findings (19). Moreover, our study extends the analysis to two other SARS-CoV-2 entry genes, TMPRSS2 and cathepsin L. Unexpectedly, smoking exerts antagonistic effects on the expression of TMPRSS2 and cathepsin L in airway epithelial cells by increasing the expression of the former but decreasing the latter. Accordingly, it is conceivable that smoking might enhance the entry of SARS-CoV-2 via membrane fusion but decrease the entry via endocytosis. In several recent meta-analysis it was reported that smoking is associated with an approximately 2 fold increase in the risk of developing severe COVID-19 (17, 56, 57), and it has been hypothesized that this increase may be caused by an elevated expression of ACE2 in smoking subjects (19). Therefore, cigarette smoking-associated changes in the expression of SARS-CoV-2 entry genes might explain the COVID-19-promoting effect.

Irrespective of the unclear pathomechanism of smoking on SARS-CoV-2 infection, our study uncovered an unexpected difference in the expression of the three genes between current smokers and former smokers, but not between former smokers and never smokers. This result indicates an enormous acute effect of smoking on the expression of SARS-CoV-2 entry genes. In contrast to many chronic and irreversible changes in the lung, smoking cessation could be effective to reduce the potential risk emanating from the alternation of the viral entry associated genes.

According to our findings, COPD, a chronic respiratory disease mainly caused by cigarette smoking, is also associated with an increased expression of ACE2 in human airway epithelial cells. However, the effect seems largely related to smoking rather than the disease itself since (i) the differences of the gene expression become visible only between COPD patients and never-smoker healthy controls, but do not appear between COPD patients and healthy smoker controls, (ii) the variations in the expression of the three genes between COPD patients and never smoker controls follow the same pattern as that between healthy smokers and never smokers, and (iii) lung function is not correlated with the expression of the three genes. Similar to COPD, patients with lung cancer also do not show higher levels of the three SARS-CoV-2 related genes than healthy controls with identical smoking status. Therefore, these results indicate that the high susceptibility to severe COVID-19 in patients with COPD or lung cancer is unlikely due to the alternations in expression of SARS-CoV-2 entry genes.

Although this study provides an overview of the expression profile of SARS-CoV-2 entry genes in human airway epithelial cells in healthy smoker and patients, three major limitations of the study need to be mentioned. First of all, this study only investigated the gene expression at the mRNA level, without providing protein data. Secondly, since the gene expression were detected by microarray, it is not possible to distinguish transcripts of a gene generated by alternative splicing. Finally, the definition of pathological conditions including COPD and lung cancer in different datasets was not always consistent, which might also affect the findings in the current study.

In conclusion, the results from this study implicate that cigarette smoking affect the expression of SARS-CoV-2 entry genes, and thus might contribute to the development of COVID-19. However, this potential risk could be reduced by smoking cessation.

## Data Availability Statement

The datasets generated for this study can be found in online repositories. The names of the repository/repositories and accession number(s) can be found in the article/Supplementary Material.

## Author Contributions

XY: study design. JY: literature search and data analysis. BK, FP, and XY: data interpretation and writing. All authors contributed to the article and approved the submitted version.

## Conflict of Interest

The authors declare that the research was conducted in the absence of any commercial or financial relationships that could be construed as a potential conflict of interest.
