# Supplementary material for: Association of Cigarette Smoking, COPD, and Lung Cancer With Expression of SARS-CoV-2 Entry Genes in Human Airway Epithelial Cells
Source: Front Med (Lausanne). 2020 Dec 4;7:619453. doi: 10.3389/fmed.2020.619453 (PMC7793919; doi:10.3389/fmed.2020.619453)
Supplement: Supplementary file 1 [file Data_Sheet_1.docx]

**Supplementary information**

**Supplementary table 1: List of datasets recruited in this study**

| **Datasets** | **Platform** | **Ref. (PMID)** | **Studying focus** | **Submitted time (year)** | **Cell type** | **Sample sizes** |
| --- | --- | --- | --- | --- | --- | --- |
| Crystal et al. Group 01 (GSE4498, GSE5058, GSE5059, GSE7832, GSE8545, GSE10006, GSE10135, GSE11784, GSE11906, GSE11952, GSE13931, GSE13933, GSE17905, GSE18385, GSE19407, GSE19667, GSE20250, GSE20257) | GPL570 [HG-U133_Plus_2] | 18339782, 17108109, 18832735, 21829517, 19852842, 20693378 | Smoking and COPD | 2006-2010 | AEC | 49 COPD, 124 NS ,167 CS |
| Crystal et al. Group 02 (GSE22047, GSE24337, GSE30063, GSE34450, GSE43939, GSE53537, GSE63127, GSE64614, GSE76324, GSE77658) | GPL570 [HG-U133_Plus_2] | 21325429, 22855713, 24465567, 26927796 | Smoking and COPD | 2010-2016 | AEC | 36 COPD, 98 NS, 129 CS |
| GSE994 | GPL96 [HG-U133A] | 15210990 | Smoking | 2004 | BEC | 34 CS, 18 FS, 23 NS |
| GSE4115 | GPL96 [HG-U133A] | 17334370 | Lung cancer | 2006 | BEC | 102 LC, 90 NC |
| GSE4302 | GPL570 [HG-U133_Plus_2] | 17898169 | Smoking | 2006 | BEC | 16 smoker, 28 HC |
| GSE7895 | GPL96 [HG-U133A] | 17894889 | Smoking | 2007 | BEC | 52 CS, 31 FS, 21 NS |
| GSE8987 | GPL571 [HG-U133A_2] | 18513428 | Smoking | 2007 | NEC | 7 CS, 8 NS |
| GSE14633 | GPL5175 [HuEx-1_0-st] | 19168627 | Smoking | 2009 | BEC | 11 CS, 11 NS |
| GSE19027 | GPL96 [HG-U133A] | 20689807 | Smoking and lung cancer | 2009 | BEC | CA: 9 CS, 12 FS; HC: 20 CS 9 FS 9 NS |
| GSE28835 | GPL13447 [HG-U133A_2] | 21636547 | Lung cancer | 2011 | LAEC | 8 LC ,5 NC |
| GSE37147 | GPL13243 [HuGene10stv1_Hs_ENSG] | 23471465 | COPD | 2012 | BEC | COPD: 30 CS, 57 FS; HC: 69 CS, 82 FS |
| GSE54495 | GPL570 [HG-U133_Plus_2] | 25705890 | Lung cancer | 2014 | Peripheral AEC | 17 LC, 13 Smoker |
| GSE56341 | GPL6244 [HuGene-1_0-st] | 24298892 | COPD | 2014 | Small AEC | 8 COPD, 14 FS |
| GSE66499 | GPL6244 [HuGene-1_0-st] | 25981554 | Lung cancer | 2015 | BEC | 490 LC ,190 HC |
| GSE67061 | GPL17077 [Agilent-039494] | 26308599 | Lung cancer | 2015 | AEC | 56 LC ,17 HC |
| GSE80796 | GPL6244 [HuGene-1_0-st] | 28376173 | Smoking and lung cancer | 2016 | NEC | CA: 113 CS, 196 FS; HC: 73 CS, 123 FS |
| GSE84101 | GPL570 [HG-U133_Plus_2] | 28273093 | Smoking | 2016 | Small AEC | 7 SM ,7 NS |
| GSE97010 | GPL17244 [HuGene-1_0-st] | 29932825 | Smoking | 2017 | BEC | 63 baseline ,63 post ASE |
| GSE112073 | GPL17556 [HuGene-1_0-st] | 31233743 | Smoking | 2018 | BEC | 9 CS, 21 FS |
| GSE128708 | GPL570 [HG-U133_Plus_2] | 32432483 | COPD | 2019 | Small AEC | 124 COPD smoker, 84 NS |

AEC, Airway epithelial cells; BEC, Bronchial and nasal epithelial cells; LAEC, Large airway epithelial cells; NEC, Nasal epithelial cells; NS, Never smoker; SM, Smoker; CS, Current smoker; FS, Former smoker; LC, Lung cancer; HC, Healthy control; COPD, Chronic obstractive pulmonary disease; ASE, Acute smoking exposure.


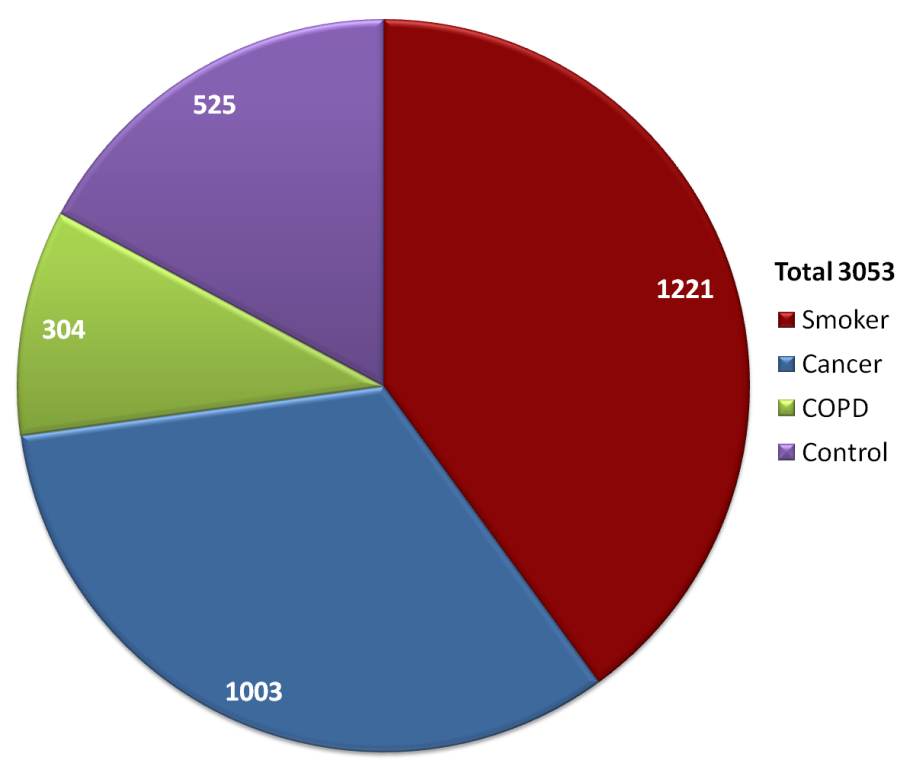


**Supplementary fig.1: Pie chart showing the composition of samples.** Control group consists of healthy never smokers and healthy subjects without information of smoking status, while the smoker group contains healthy smokers, including both current smokers and former smokers.

**Supplementary Fig. 2. Impact of age on gene expression of SARS-CoV-2 entry genes in human airway epithelial cells.** Linear regression was used to determine the relationship between age and expression of ACE2, TMPRSS2 and CTSL in never smokers, current smokers, COPD patients and patients with lung cancer. Data was retrieved from dataset Crystal01 and GSE80796. Linear regression line with 95% confidence interval (grey band), P value and Pearson correlation coefficient (R) were presented. * p < 0.05.


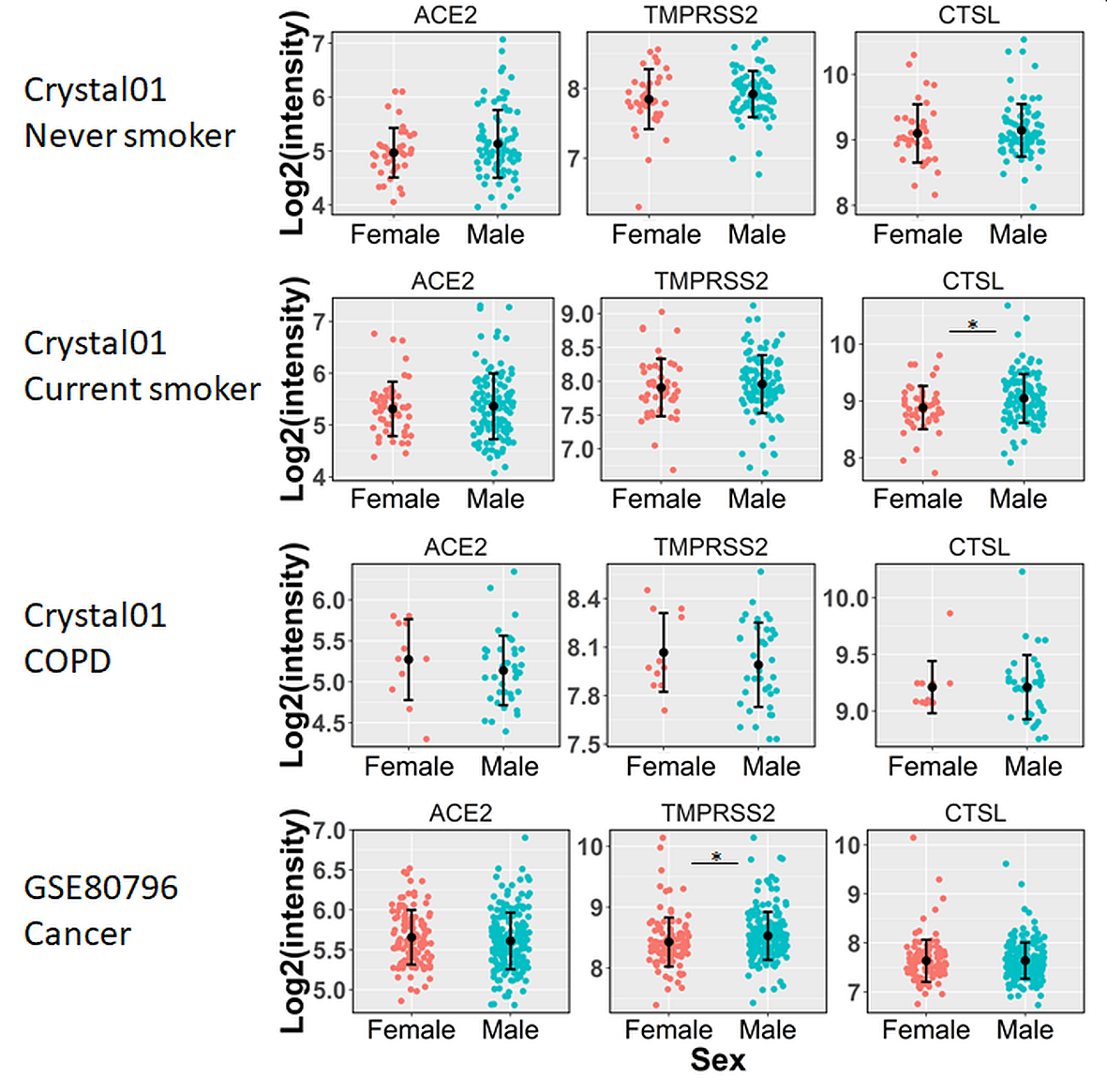


**Supplementary Fig. 3. Impact of sex on gene expression SARS-CoV-2 entry genes in human airway epithelial cells.**  Difference between male and female subjects in gene expression of ACE2, TMPRSS2 and CTSL in never smokers, current smokers, patients with COPD or lung cancer. Data was retrieved from dataset Crystal01 and GSE80796. P values were calculated by student’s t test. * p < 0.05.


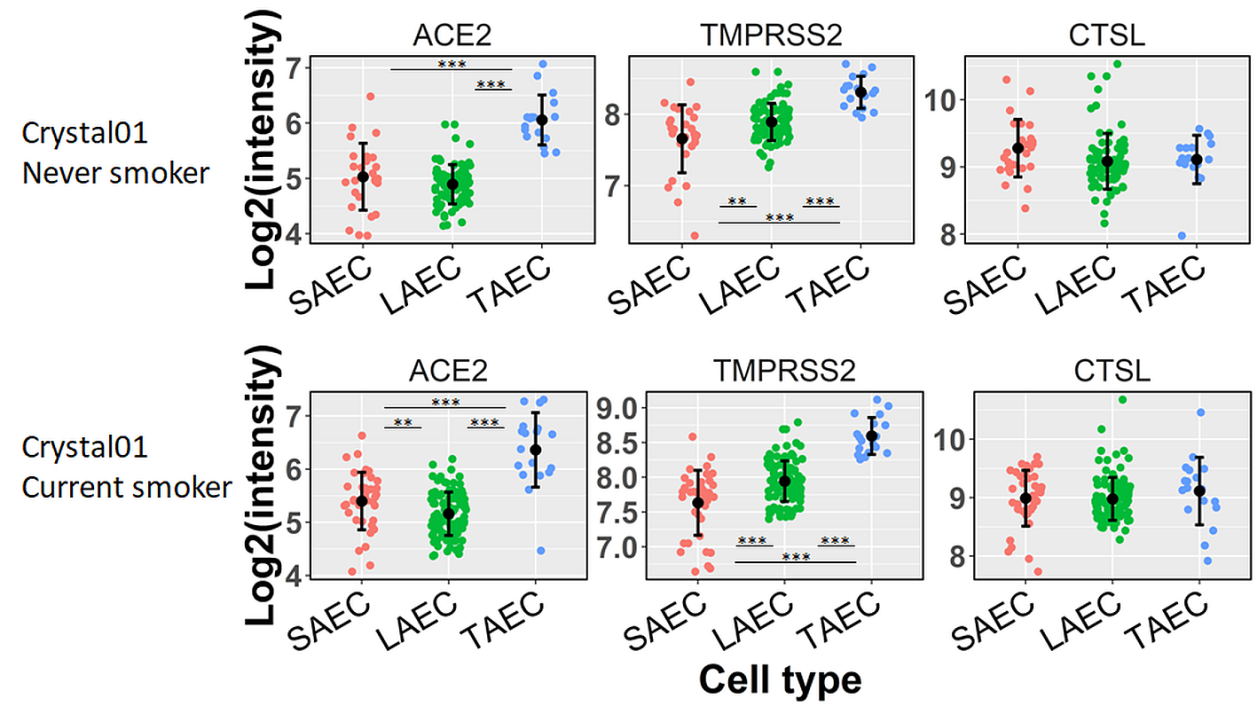


**Supplementary Fig. 4. Expression of SARS-CoV-2 entry genes in different types of human airway epithelial cells.** Difference in gene expression of ACE2, TMPRSS2 and CTSL between small airway epithelial cells (SAEC), large airway epithelial cells (LAEC) and trachea airway epithelial cells (TAEC) in subjects in never smokers and current smokers. Data was retrieved from dataset Crystal01. P values were calculated by student’s t test. ** p < 0.01, *** p < 0.001.

**Supplementary figure 5. Expression of ACE2, TMPRSS2 and cathepsin L (CTSL) in airway epithelial cells before (baseline) and 24 hours after acute smoke exposure (post-ASE) in asymptomatic smokers.** Gene expression data were retrieved from dataset GSE97010. The post-ASE group consists of 63 smokers who were asked to refrain from cigarette smoking for at least 2 days and then subjected to ASE, while the 63 subjects underwent bronchoscope at a separate time at least 6 wks from the post-smoking bronchoscope to serve as an unexposed baseline group. Statistical difference was analyzed by paired Student t test. * p<0.05.

**Supplementary figure 6. Correlation between the pack**-**years of smoking and expression of SARS-CoV-2 entry genes in airway epithelial cells in current smokers.** Forest plots of meta-analysis for correlation between the pack-years and expression of ACE2, TMPRSS2 and CTSL in airway epithelial cells of current smokers. The x-axis indicates the correlation coefficients, while the y-axis shows GEO datasets and cell types. The correlation coefficients (95% CI) and p values of meta-analysis are depicted. BEC, bronchial epithelial cells; NEC, nasal epithelial cells; SAEC, small airway epithelial cell; LAEC, large airway epithelial cell; TAEC, trachea airway epithelial cell.

**Supplementary Fig. 7. Correlation between** **SARS-CoV-2 entry genes with lung function indexes in COPD patients.** Linear regression of FEV1 (A, C, E) and FEV1/FVC (B, D, F) with expression of ACE2 (A, B), TMPRSS2 (C, D) and cathepsin L (CTSL) (E, F). Data were retrieved from dataset GSE37147. FEV1, the first second of forced expiration to the full; FVC, forced vital capacity; FEV1/FVC, ratio of FEV1 and FVC. Linear regression line and 95% confidence interval (grey band), *p* values and coefficient of determination (R^2^) calculated by using multiple linear regression model in which age was used a covariate were presented.


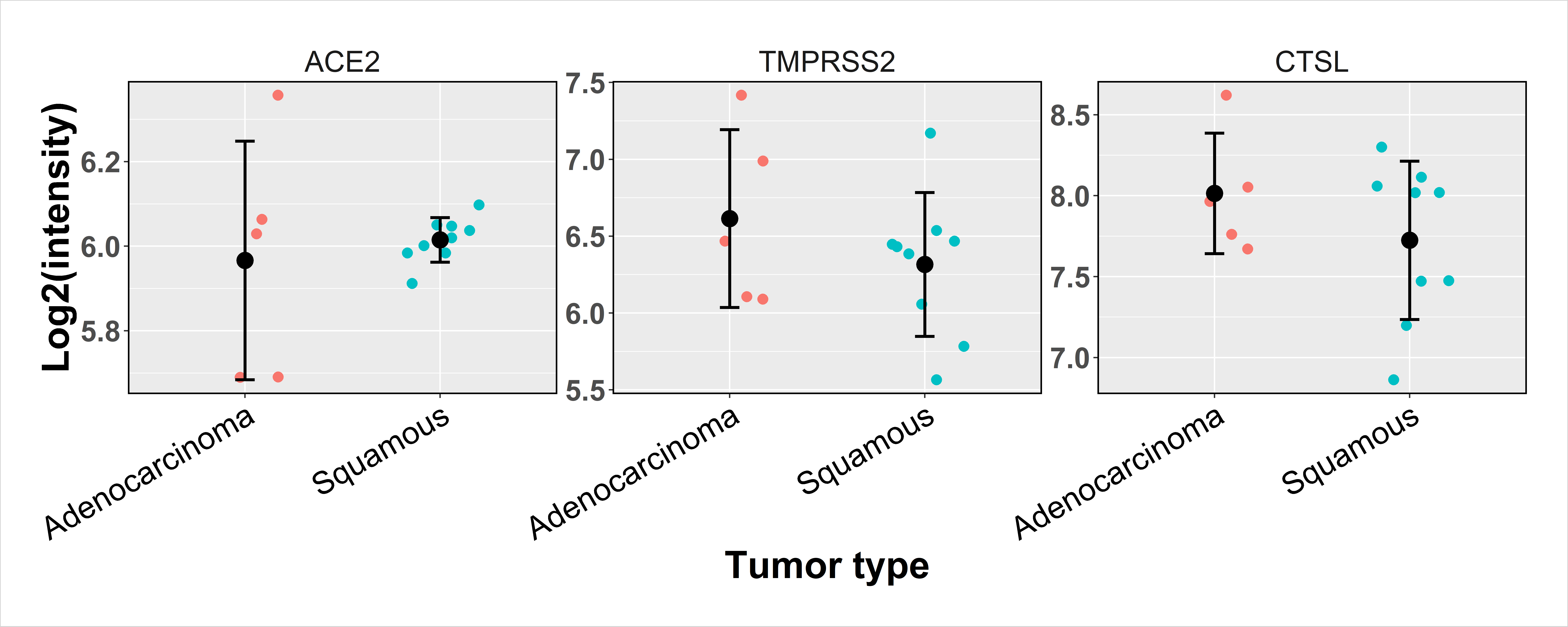


**Supplementary Fig. 8. Expression of SARS-CoV-2 entry genes in airway epithelial cells in different types of lung cancer.** Histodot plot of expression of ACE2, TMPRSS2 and CTSL in adenocarcinoma and squamous cell carcinoma. Data was retrieved from dataset GSE19027 and presented as mean ± SD (standard deviation). No significant difference was observed between the two types of lung cancer in expression of ACE2, TMPRSS2 or CTSL.

**Supplementary Fig. 9. Effect of cancer-related mutations on expression of SARS-CoV-2 entry genes in airway epithelial cells in different mutation types of lung adenocarcinoma. A.** Histodot plot of expression of ACE2, TMPRSS2 and CTSL in airway epithelial cells in adenocarcinoma patients with (Mut, n=154) or without (WT, n=288) *KRAS* mutation. B**.** Histodot plot of expression of ACE2, TMPRSS2 and CTSL in airway epithelial cells in adenocarcinoma patients with (Mut, n=47) or without (WT, n=395) *EGFR* mutation. Data was retrieved from dataset GSE72094 and presented as mean ± SD (standard deviation). *p* values were calculated by using Student’s t test or Mann Whitney test. *, p < 0.05; ** p < 0.01; ***, p < 0.001.
